# Involvement of metformin and aging in salivary expression of ACE2 and TMPRSS2

**DOI:** 10.1002/biof.2154

**Published:** 2025-01-26

**Authors:** Yosuke Shikama, Kunihiro Otsuka, Yuka Shikama, Masae Furukawa, Naozumi Ishimaru, Kenji Matsushita

**Affiliations:** ^1^ Department of Oral Disease Research National Center for Geriatrics and Gerontology Obu Japan; ^2^ Department of Geriatric Oral Science, Graduate School of Dentistry Tohoku University Sendai Japan; ^3^ Department of Oral Molecular Pathology Tokushima University Graduate School of Biomedical Sciences Tokushima Japan; ^4^ Department of Oral Pathology Graduate School of Medical and Dental Sciences, Institute of Science Tokyo Tokyo Japan

**Keywords:** ADAM17, epithelial cells, saliva, salivary glands, SARS‐CoV‐2

## Abstract

SARS‐CoV‐2‐related proteins, ACE2 and TMPRSS2, are determinants of SARS‐CoV‐2 infection. Although these proteins are expressed in oral‐related tissues, their expression patterns and modulatory mechanisms in the salivary glands remain unknown. We herein showed that full‐length ACE2, which has both a fully functional enzyme catalytic site and high‐affinity SARS‐CoV‐2 spike S1‐binding sites, was more highly expressed in salivary glands than in oral mucosal epithelial cells and the lungs. Regarding TMPRSS2, zymogen and the cleaved form were both expressed in the salivary glands, whereas only zymogen was expressed in murine lacrimal glands and the lungs. Metformin, an AMPK activator, increased stimulated saliva secretion and full‐length ACE2 expression and decreased cleaved TMPRSS2 expression in the salivary glands, and exerted the same effects on soluble ACE2 (sACE2) and sTMPRSS2 in saliva. Moreover, metformin decreased the expression of beta‐galactosidase, a senescence marker, and ADAM17, a sheddase of ACE2 to sACE2, in the salivary glands. In aged mice, the expression of ACE2 was decreased in the salivary glands, whereas that of sACE2 was increased in saliva, presumably by the up‐regulated expression of ADAM17. The expression of TMPRSS2 in the salivary glands and sTMPRSS2 in saliva were both increased. Collectively, these results suggest that the protein expression patterns of ACE2 and TMPRSS2 in the salivary glands differ from those in other oral‐related cells and tissues, and also that metformin and aging affect the salivary expression of ACE2 and TMPRSS2, which have the potential as targets for preventing the transmission of SARS‐CoV‐2.

## Graphical abstract

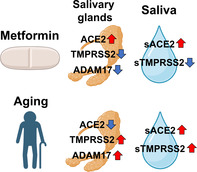

## 1 INTRODUCTION

Severe acute respiratory syndrome coronavirus 2 (SARS‐CoV‐2), a single‐stranded positive‐strand RNA virus that belongs to lineage B, clade 1 of the beta coronavirus genus,^1^ is the causal agent for coronavirus disease 2019 (COVID‐19) and its transmission occurs via both saliva droplets and aerosols.^2^ The virus binds to host cells through its spike (S) glycoproteins, which consist of S1 and S2 subunits. The S1 subunit contains a receptor‐binding domain (RBD) that recognizes and binds to host cells, while the S2 subunit mediates viral cell membrane fusion.^3^ Angiotensin‐converting enzyme 2 (ACE2) is a lineage B clade 1‐specific receptor including SARS‐CoV‐2. Since the RBD of the S1 subunit directly binds to ACE2, it is the most important targeting site to inhibit viral infection.^1^ Furthermore, transmembrane protease serine 2 (TMPRSS2) is used for S protein activation, thereby strengthening the binding of SARS‐CoV‐2 and ACE2; therefore, the invasion of SARS‐CoV‐2 can be blocked by TMPRSS2 inhibitors.^4^ Since ACE2 and TMPRSS2 are both expressed in oral‐related tissues, such as the tongue epithelium, gingival tissues, and salivary glands,^2^, ^5^ these host entry factors have potential as targets for preventing SARS‐CoV‐2 infection and its spread via the oral cavity.

The expression of ACE2 is regulated by physiological and pathological conditions, such as diabetes and aging,^6^ and also by adenosine monophosphate‐activated protein kinase (AMPK).^7^, ^8^ AMPK plays a key role as a master regulator of cellular energy homeostasis, and its dysregulation has been reported in chronic low‐grade inflammatory conditions, including obesity, diabetes, and aging.^9^ ACE2 is cleaved to the soluble form of ACE2 (sACE2) by host proteases, such as a disintegrin and metalloprotease 17 (ADAM17),^10^ which is released in body fluids, such as saliva.^11^ Recombinant sACE2 is used to neutralize SARS‐CoV‐2 as a decoy receptor, and its therapeutic potency was confirmed using human organoids^12^ and in a clinical trial.^13^ Moreover, the soluble form of TMPRSS2 (sTMPRSS2) has been detected in saliva.^14^

Metformin, a clinically available AMPK activator, is a well‐known drug prescribed for patients with type II diabetes to decrease glucose absorption, improve peripheral glucose uptake, and increase insulin sensitivity, thereby reducing blood glucose levels.^15^ In addition to these effects, previous studies demonstrated the potential of metformin as an anti‐cancer and anti‐aging agent.^16^ Since metformin may affect age‐related changes in the salivary expression of ACE2 and TMPRSS2, we herein investigated whether metformin and aging modulated the expression of these proteins in salivary glands and saliva. The results obtained revealed the presence of the full‐length ACE2 protein, which has the binding site for the S1 protein, and truncated TMPRSS2, which has a trypsin‐like S1 peptidase domain,^17^ in the salivary glands. Moreover, a treatment with metformin increased the salivary expression level of ACE2 without affecting its serum level, and decreased that of TMPRSS2, which is the cleaved protease domain fragment. Although the expression level of the ACE2 protein was decreased in the salivary glands of aged mice, the protein level of sACE2 was significantly increased in the saliva of aged mice, presumably via the up‐regulation of ADAM17 in the salivary glands of aged mice. The expression level of TMPRSS2 was significantly increased in both the salivary glands and saliva of aged mice.

## 2 METHODS

### 2.1 Mice, metformin treatment, and saliva collection

All animal experiments were approved by and conducted in accordance with the guidelines established by the National Center for Geriatrics and Gerontology Animal Ethics Committee (4‐19, 3/3/2022). Experienced researchers performed the animal protocol in accordance with the ARRIVE 2.0 guidelines. Young C57BL/6N male mice (age: 6 weeks) and aged adult C57BL/6N male mice (age: 18–22 months) were obtained from Japan SLC Inc. (Hamamatsu, Japan) or the Experimental Animal Facility at the National Center for Geriatrics and Gerontology (Obu, Japan), respectively. Mice were housed in specific pathogen‐free conditions under a 12‐h light–dark photocycle. They had ad libitum access to water and food and were in plastic cages with an internal area of 384 cm^2^ × 14 cm depth, with paper bedding, up to a maximum of five animals per cage. The temperature in the room was maintained at 23 ± 2°C and humidity at 50 ± 10%.

Young mice were randomly divided into the Control (water) and Metformin (Wako Pure Chemical Industries, Tokyo, Japan) groups. Metformin was orally administered via drinking water at a 5 mg/mL dose to mice starting at 6 weeks old. These doses were selected based on those used in other studies on mice.^18^, ^19^

Saliva stimulated by pilocarpine was collected and its volume was measured as previously described.^20^ Collected saliva was immediately cooled on ice, and collected samples were centrifuged at 3000*g* at 4°C for 5 min. The supernatant was frozen at −80°C until analyzed.

### 2.2 RNA isolation and a quantitative real‐time PCR analysis

Total RNA was extracted from cells using the RNeasy mini kit (Qiagen, Hilden, Germany) according to the manufacturer's instructions. Total RNA concentrations were measured using a Nanodrop spectrophotometer (Thermo Fisher Scientific, Waltham, MA, USA), and cDNA was synthesized with the PrimeScript RT Master Mix (TaKaRa Bio Inc., Shiga, Japan). qRT‐PCR was performed on a LightCycler 96 system using FastStart Essential DNA Green Master (Roche Applied Science, Mannheim, Germany). The following primers were used for the amplification of specific genes: *Ace2*, 5′‐TGATGAATCAGGGCTGGGATG‐3′ (sense) and 5′‐ATTCTGAAGTCTCCGTGTCCC‐3′ (antisense), *Tmprss2*, 5′‐GAGAACCGTTGTGTTCGTCTC‐3′ (sense) and 5′‐GCTCTGGTCTGGTATCCCTTG‐3′ (antisense), *Adam17*, 5′‐TGTGGTTATTTAAATGCAGATAGTGA‐3′ (sense) and 5′‐TCACTCGACGAACAAACTCTTC (antisense), *Cdkn2a* (*p16* ^*INK4a*^), 5′‐CGTACCCCGATTCAGGTGAT (sense) and 5′‐TTGAGCAGAAGAGCTGCTACGT (antisense), and *Gapdh*, 5′‐GCCTTCCGTGTTCCTACCC‐3′ (sense) and 5′‐TGAAGTCGCAGGAGACAACC‐3′ (antisense). The relative quantification of gene expression was performed according to the 2^−ΔΔCT^ method and normalized against *Gapdh* mRNA.

### 2.3 Cells and cell culture

The A‐253 human submandibular gland carcinoma cell line (HTB‐41) and human primary gingival keratinocytes (HGK, PCS‐200‐014) were purchased from the American Type Culture Collection and cultured in McCoy's 5A (Modified) Medium supplemented with 10% FBS, 100 U/mL penicillin, and 100 μg/mL streptomycin, or KGM‐Gold™ BulletKit™ (Lonza Japan, Tokyo, Japan), respectively. Human oral keratinocytes (HOK) isolated from the oral mucosa were purchased from ScienCell Research Laboratories (San Diego, CA) and cultured in KGM‐Gold™ BulletKit™. The HSC‐2 human oral squamous carcinoma cell line was provided by the Japanese Collection of Research Bioresources Cell Bank (Osaka, Japan) and cultured in DMEM supplemented with 10% FBS, 100 U/mL penicillin, and 100 μg/mL streptomycin. These cells were cultured at 37°C in a humidified atmosphere of 5% CO_2_.

### 2.4 Western blot analysis

Cultured cells were lysed in RIPA buffer supplemented with a 10% protease and phosphatase inhibitor cocktail (Thermo Fisher Scientific). Murine tissues were lysed in T‐PER™ Tissue Protein Extraction Reagent supplemented with 20% protease and the phosphatase inhibitor cocktail using the Miltenyi gentleMACS Octo Dissociator following the preset Protein_01_01 program. Lysates were centrifuged at 12,000g at 4°C for 10 min and the supernatants were collected. The protein concentrations of lysates and saliva were assessed using the BCA protein assay kit (Thermo Fisher Scientific). Human salivary gland (hSG) extract was purchased from Santa Cruz Biotechnology (sc‐363762, Santa Cruz, CA, USA). Protein concentrations were adjusted, and samples were then diluted in 2× or 4× Laemmli Sample Buffer. After boiling at 95°C for 5 min, proteins were separated using sodium dodecyl sulfate‐polyacrylamide gel electrophoresis and transferred to polyvinylidene difluoride membranes (Bio‐Rad, Hercules, CA, USA). Membranes were incubated with antibodies against ACE2 (for tissue lysates: AF3437, for saliva: AF933, R&D Systems, Minneapolis, MN, USA), α‐amylase (A8273, Sigma‐Aldrich, St. Louis, MO, USA), ADAM17 (GTX101358, GeneTex, Alton Pkwy Irvine, CA, USA), GAPDH (5174, Cell Signaling Technology, Danvers, MA, USA), TMPRSS2 (for human samples: sc‐515727, Santa Cruz Biotechnology, for murine samples: bs‐6285R, Bioss, Woburn, MA, USA), aquaporin 5 (AQP5) (AQP‐005, Alomone Labs, Jerusalem, Israel), α‐tubulin (3873, Cell Signaling Technology), cytokeratin 19 (CK19) (MABT913, Sigma‐Aldrich), and p21 Waf1/Cip1 (64016, Cell Signaling Technology).

Antibody dilutions were adjusted according to the manufacturer's instructions. Proteins were visualized with Immunostar (Wako) and Amersham Imager 680, and the optical densities of protein bands were measured with Amersham Imager 680 Analysis Software (GE Healthcare, Piscataway, NJ, USA).

### 2.5 Sample preparation for immunohistochemical staining

Archived paraffin‐embedded tissue specimens were used. Tissue sections were deparaffinized in xylene and rehydrated in descending grades of ethanol. Antigen retrieval was performed using a pressure cooker and citrate phosphate buffer (pH 6.0). Endogenous peroxidase activity was blocked with 3% H_2_O_2_ for 10 min. Sections were treated with normal rabbit IgG (148‐09551, Wako), normal goat IgG (AB‐108‐C, R&D Systems), anti‐ACE2 (AF3437, R&D Systems), ADAM17 (GTX101358, GeneTex), beta‐galactosidase (15518‐1‐AP, ProteinTech, Rosemont, IL, USA), or TMPRSS2 (bs‐6285R, Bioss) antibodies at 4°C overnight. Antibody dilutions were adjusted according to the manufacturer's instructions. After washing with PBS, sections were treated with TaKaRa POD Conjugate Anti Rabbit For Mouse Tissue (MK202, TaKaRa) or ImmPRESS Reagent Anti‐Goat Ig (MP‐7405, Vector Laboratories, Newark, CA, USA). The reaction was detected by diaminobenzidine (MK210, TaKaRa) staining. Sections were then counterstained with hematoxylin, dehydrated in ascending grades of ethanol, and mounted on slides. Positive areas in sections were measured using OLYMPUS cellSens Imaging Software (Version 4.1.1). We confirmed that the value of isotype images was zero for quantification.

### 2.6 Enzyme‐linked immunosorbent assay

The serum concentration of ACE2 was measured in mice using Mouse ACE‐2 DuoSet ELISA (DY3437‐05, R&D Systems). Briefly, 96‐well flat‐bottomed plates were precoated with capture antibodies, and diluted samples and standard recombinant ACE2 were added to each well. After the plates were washed, biotinylated antibodies were added, and the wells were incubated with horseradish peroxidase‐labeled streptavidin. The mixture of TMB Substrate Reagent (555214, BD San Diego, CA, USA) was added to each well as the substrate. Optimal density at 450 nm was measured using a microplate reader (51119050, Thermo Fisher Scientific).

### 2.7 Isolation of epithelial cells from salivary glands using magnetic cell sorting

Murine salivary glands were minced and dissociated in a gentleMACS C tube with 2.5 mL of a digestion cocktail prepared with Multi Tissue Dissociation Kit 1 (#130‐110‐201, Miltenyi Biotech, Auburn, CA) in RPMI 1640. Digestion was performed in a Miltenyi gentleMACS Octo Dissociator with Heaters following the preset 37C_mSG_1 program. Ten milliliters of 0.5% BSA/2 mM EDTA/PBS was added to dissociated cells and filtered through a 100‐μm strainer. The cell suspension was centrifuged at 300g at 4°C for 10 min, the supernatant was removed, and cells were resuspended in 0.5% BSA/2 mM EDTA/PBS. MSEC from the cell suspension were collected by negative selection using Miltenyi mouse CD45 MicroBeads (130‐052‐301) and positive selection using Miltenyi mouse CD326 (epithelial cell adhesion molecule) MicroBeads (130‐105‐958) according to the manufacturer's instructions.

### 2.8 Primary culture of MSEC

In Defined Keratinocyte‐SFM (Gibco, Carlsbad, CA, USA) supplemented with 10 μM Y‐27632 (Fujifilm Wako, Saitama, Japan),^21^ isolated MSEC described above were cultured in collagen type I‐coated dishes or well plates (AGC TECHNO GLASS, Shizuoka, Japan). To induce replicative senescence, cells were seeded at 3 × 10^5^ cells in 60‐mm dishes, cultured until subconfluent, and subcultured repeatedly to the 15th generation (passage 15). Regarding the metformin treatment, cells (passage 1) were seeded at 1 × 10^6^ cells per well in 6‐well plates and incubated with various metformin concentrations for 48 h. Metformin was dissolved in phosphate‐buffered saline (PBS) as a stock solution of 1 M.^22^

### 2.9 Data and statistical analyses

Data from tissues and saliva of aged mice that had obvious malignant tumors were excluded. The investigators were not blinded during data collection. Based on our previous published studies, the sample size in the present study was adequately powered to detect the observed differences between the experimental groups. The declared group size is the number of independent values, and the statistical analysis was performed using these independent values. The significance of differences was evaluated using the Student's unpaired *t*‐test or Dunnett's multiple comparison test after an analysis of variance using GraphPad Prism (version 10.0.1 GraphPad Software). Values of *p* < 0.05 were considered to be significant.

## 3 RESULTS

### 3.1 Expression pattern of the ACE2 protein in human and murine salivary glands and oral‐related cells: effects of the metformin treatment on its expression

**FIGURE 1:**
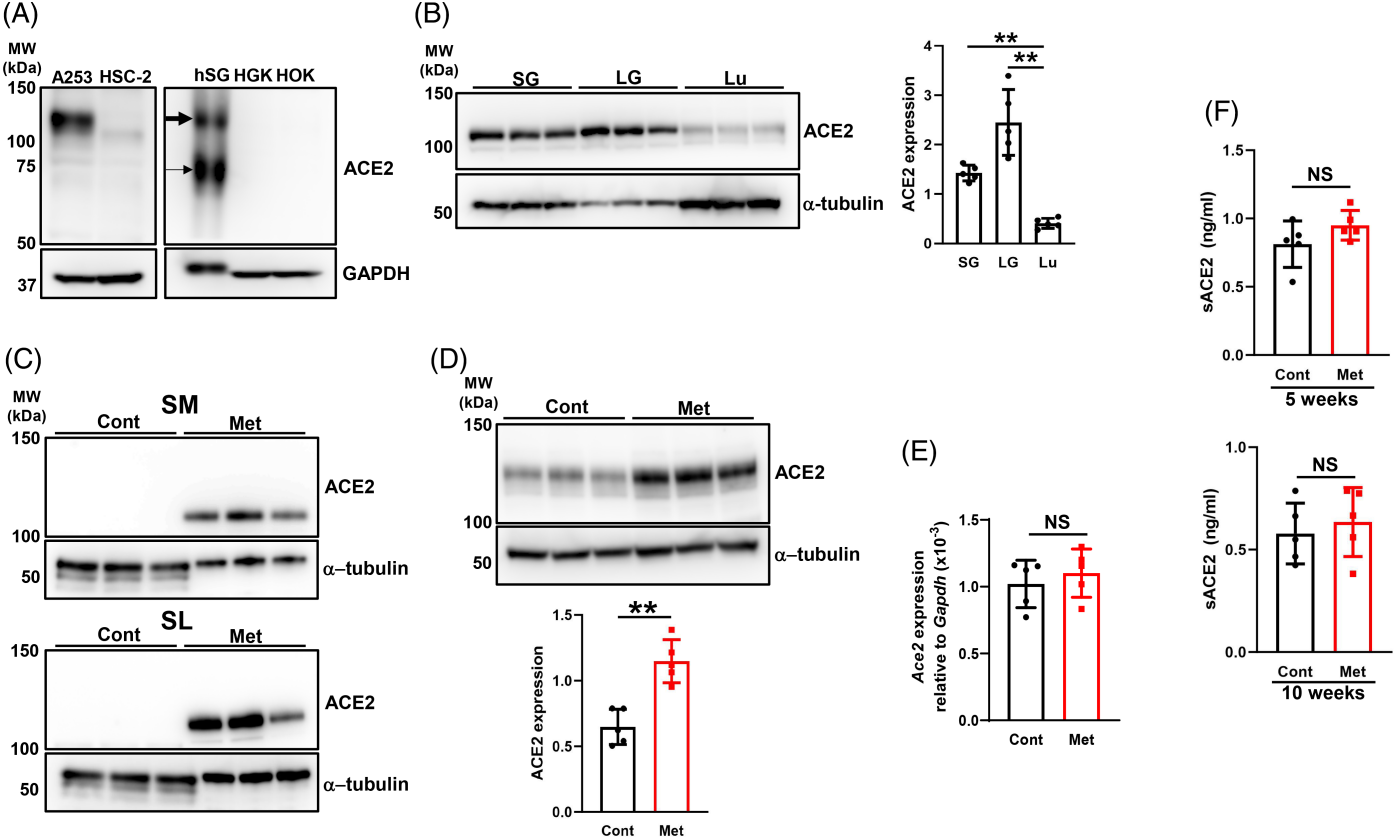
Full‐length ACE2 expression in salivary glands and effects of metformin on its expression. (A) Whole‐cell lysates prepared from human salivary glands (hSG) and human salivary and oral‐related cells were immunoblotted with anti‐ACE2 and anti‐GAPDH antibodies. Representative blots from three independent experiments are shown. Thick and thin arrows indicate full‐length ACE2 (fACE2) and the truncated ACE2 protein, respectively. (B) Whole‐cell lysates prepared from the salivary glands (SG: submandibular and sublingual glands), lacrimal glands (LG), and lungs (Lu) of young mice (*N* = 3) were immunoblotted with anti‐ACE2 and anti‐α‐tubulin antibodies. The bar graph shows integrated signal intensities in fACE2 normalized to that of α‐tubulin (*N* = 5). (C) Whole‐cell lysates prepared from the submandibular glands (SM) and sublingual glands (SL) of control (Cont) mice and mice treated with metformin (Met) (*N* = 3 each) for 8 weeks were immunoblotted with anti‐ACE2 and anti‐α‐tubulin antibodies. Representative blots from two independent experiments are shown. (D) Whole‐cell lysates prepared from the lungs of Cont mice and mice treated with Met (*N* = 3 each) for 8 weeks were immunoblotted with anti‐ACE2 and anti‐α‐tubulin antibodies. The bar graph shows integrated signal intensities for fACE2 normalized to that of α‐tubulin (*N* = 5). (E) *ACE2* mRNA expression levels in epithelial cells of the salivary glands of Cont mice and mice treated with Met (*N* = 5 each) for 10 weeks. (F) Detection of sACE2 by ELISA in serum collected from Cont mice and mice treated with Met (*N* = 5 each) for the indicated periods. Values are presented as means ± standard deviations (SD). NS, not significant. **p < 0.01 (the unpaired Student's *t*‐test). A253, a human submandibular gland carcinoma cell line; HSC‐2, a human oral squamous carcinoma cell line; HGK, human primary gingival keratinocytes; HOK, human primary oral keratinocytes.

Recent studies demonstrated the presence of the truncated ACE2 isoform, which lacks both a fully functional enzyme catalytic site and high affinity spike S1‐binding sites, in epithelial tissues.^23^, ^24^ Therefore, we investigated the expression pattern of ACE2 in cell lines and primary cells derived from salivary glands and the oral mucosa. The expression of full‐length ACE2 (~100 kDa) (fACE2) was higher in cell lysates collected from A253 cells than from HSC‐2 cells. Moreover, although fACE2 has been detected in human gingival epithelial cells,^25^ the expression levels of both the full‐length and truncated (~75 kDa)^23^, ^26^ ACE2 protein were markedly higher in hSG than in HGK and HOK (Figure 1A), suggesting that epithelial cells of hSG are more susceptible to binding to the S1 protein than oral mucosal epithelial cells. We then examined the expression levels of fACE2 in murine salivary glands, lacrimal glands, and the lungs using Western blotting. We already confirmed the expression of ACE2 and TMPRSS2 in serous acinar cells and ductal epithelial cells of the salivary glands by immunohistochemical staining (IHC) (Figure S1). Interestingly, fACE2 expression levels were significantly higher in the salivary glands and lacrimal glands than in the lungs (Figure 1B). To examine the effects of the metformin treatment on fACE2 expression in the salivary glands, young mice were aged in‐house for 8 weeks with or without metformin in their drinking water. fACE2 expression levels were markedly increased in the submandibular and sublingual glands of mice treated with metformin (Figure 1C). Moreover, its expression levels were significantly increased in the lungs of mice treated with metformin (Figure 1D). However, no significant differences were observed in ACE2 mRNA expression levels in epithelial cells of the salivary glands between control and metformin‐treated mice (Figure 1E) or in serum sACE2 levels between control and metformin‐treated mice (Figure 1F). These results indicate that fACE2 was expressed in the salivary glands, its expression levels were higher in the salivary glands than in the lungs, and the metformin treatment increased fACE2 expression levels in the salivary glands without affecting ACE2 mRNA expression levels in the salivary glands or serum sACE2 levels.

### 3.2 Expression pattern of the TMPRSS2 protein in human and murine salivary glands and oral‐related cells: effects of the metformin treatment on its expression

**FIGURE 2:**
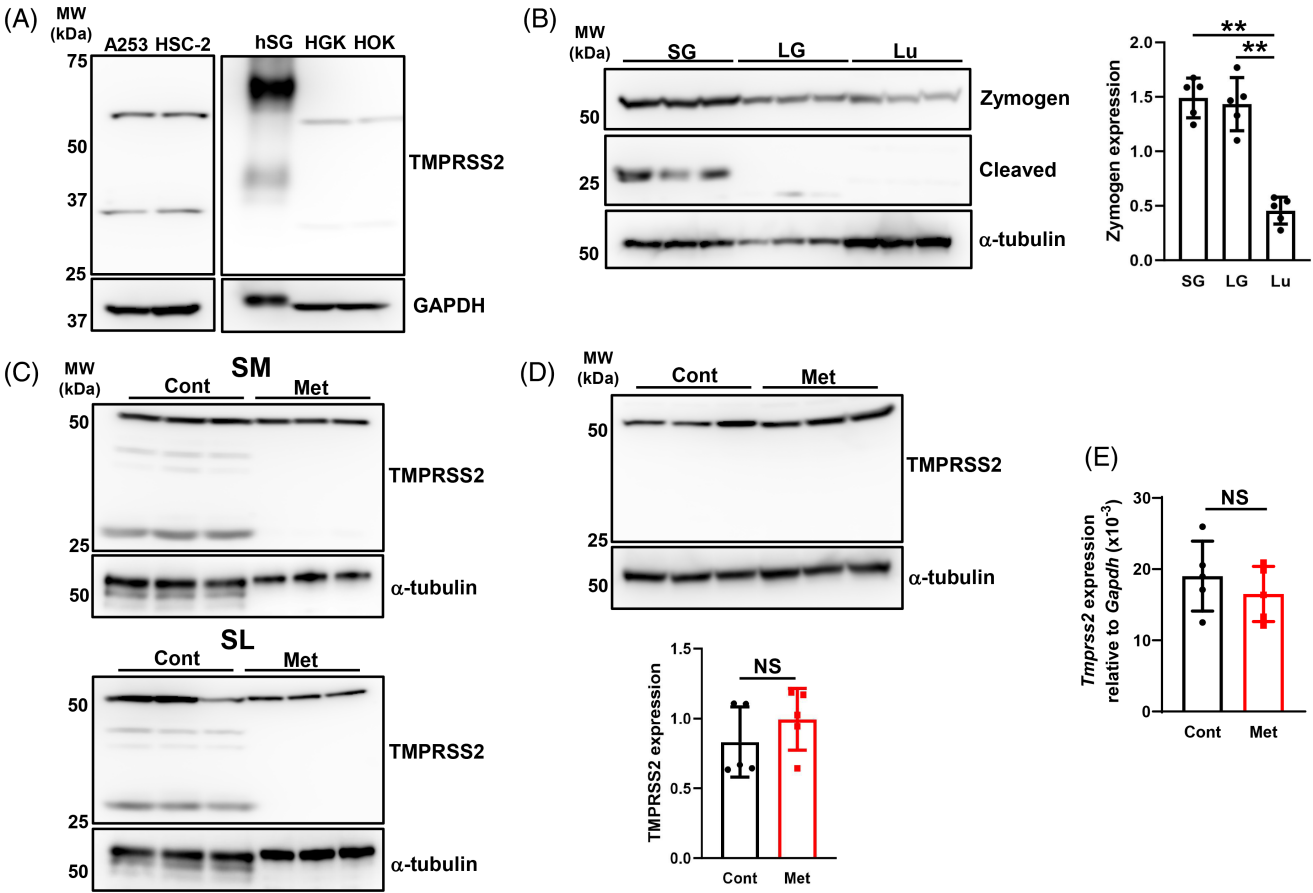
TMPRSS2 expression in salivary glands and effects of metformin on its expression. (A) Whole‐cell lysates prepared from human salivary glands (hSG) and human salivary and oral‐related cells were immunoblotted with anti‐TMPRSS2 and anti‐GAPDH antibodies. Representative blots from three independent experiments are shown. (B) Whole‐cell lysates prepared from the salivary glands (SG, submandibular and sublingual glands), lacrimal glands (LG), and lungs (Lu) of young mice (*N* = 3) were immunoblotted with anti‐TMPRSS2 and anti‐α‐tubulin antibodies. The bar graph shows integrated signal intensities for TMPRSS2 zymogen normalized to that of α‐tubulin (*N* = 5). (C) Whole‐cell lysates prepared from the submandibular glands (SM) and sublingual glands (SL) of control (Cont) mice and mice treated with metformin (Met) (*N* = 3 each) for 8 weeks were immunoblotted with anti‐TMPRSS2 and anti‐α‐tubulin antibodies. Representative blots from two independent experiments are shown. (D) Whole‐cell lysates prepared from the lungs of Cont mice and mice treated with Met (*N* = 3 each) for 8 weeks were immunoblotted with anti‐TMPRSS2 and anti‐α‐tubulin antibodies. The bar graph shows integrated signal intensities for TMPRSS2 normalized to that of α‐tubulin (*N* = 5). (E) *TMPRSS2* mRNA expression levels in the epithelial cells of the salivary glands of Cont mice and mice treated with Met (*N* = 5 each) for 10 weeks. Values are presented as means ± SD. NS, not significant. **p < 0.01 (the unpaired Student's *t*‐test). A253, a human submandibular gland carcinoma cell line; HSC‐2, a human oral squamous carcinoma cell line; HGK, human primary gingival keratinocytes; HOK, human primary oral keratinocytes. Images detecting GAPDH and α‐tubulin proteins are identical, as shown in Figure 1.

The calculated molecular weight of TMPRSS2 protein is approximately 54 kDa, which is a zymogen, whereas native and recombinant human TMPRSS2 proteins have a higher molecular mass (~60–70 kDa) due to N‐linked glycosylation. In lysates collected from A253, HSC‐2, HGK, and HOK, we detected bands at 65 and 31–35 kDa, representing the zymogen and cleaved protease domain fragment, respectively.^17^, ^27^ However, in hSG lysates, TMPRSS2 was detected as two protein bands at 70 kDa (major band) and 40 kDa (minor band), with the minor band potentially representing a proteolytic degradation product^27^ (Figure 2A). In murine tissues, the zymogen was detected at 54 kDa, and expression levels were significantly higher in the salivary glands and lacrimal glands than in the lungs. The trypsin‐like S1 peptidase domain, 25‐kDa cleaved TMPRSS2,^28^ was only detected in the salivary glands (Figure 2B). Moreover, its expression level was markedly decreased in the submandibular and sublingual glands of metformin‐treated mice (Figure 2C). The metformin treatment did not affect the expression levels of the zymogen or cleaved form of TMPRSS2 in the lungs (Figure 2D). Similar to ACE2, no significant differences were observed in TMPRSS2 mRNA expression levels between epithelial cells in the salivary glands of control and metformin‐treated mice (Figure 2E). These results suggest that the cleaved form of TMPRSS2 was constitutively expressed and its expression level was decreased by the metformin treatment without affecting TMPRSS2 mRNA expression levels in the salivary glands.

### 3.3 Effects of the metformin treatment on senescence‐associated beta‐galactosidase and ADAM17 expression in murine salivary glands

**FIGURE 3:**
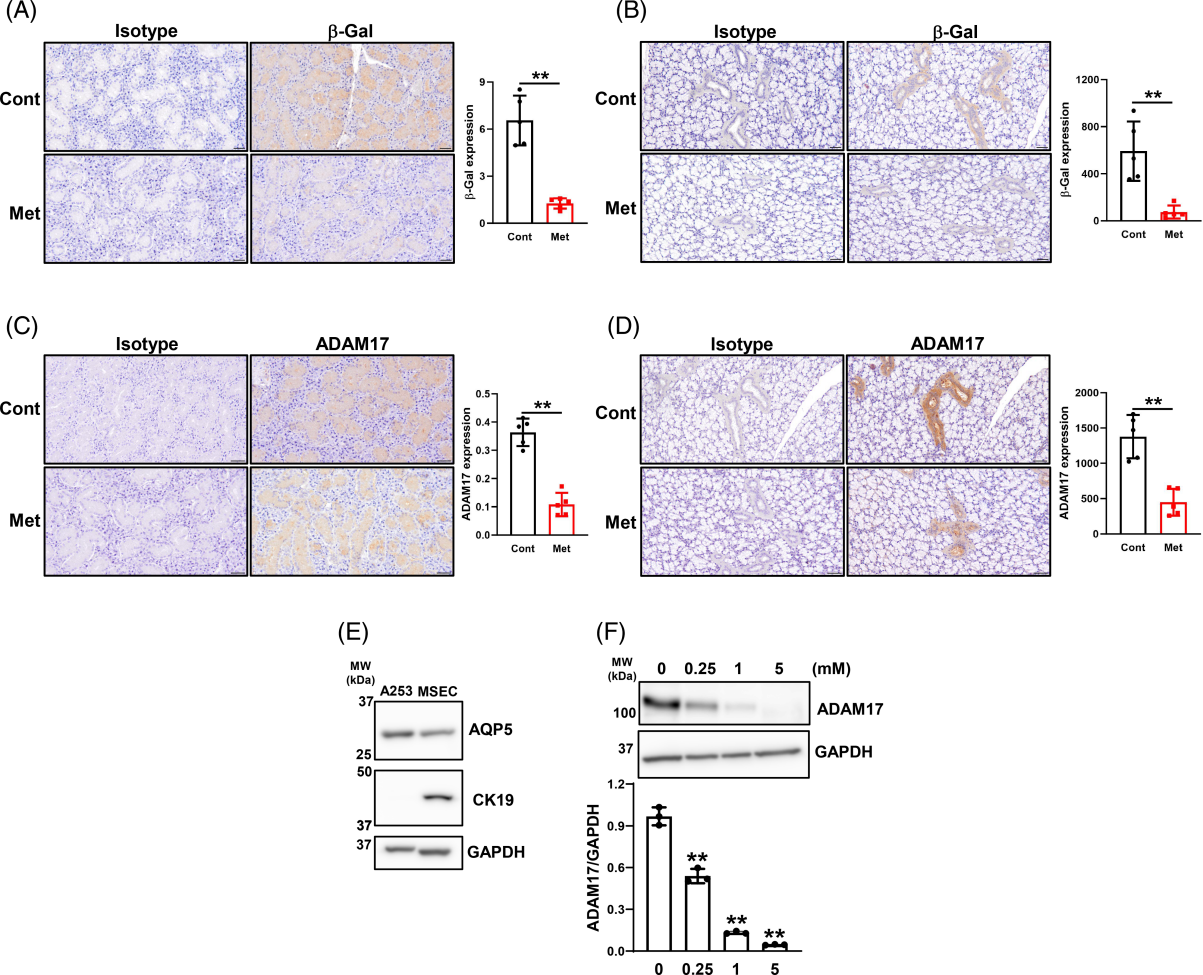
Decreases in β‐Gal and ADAM17 expression by metformin in murine salivary glands. (A, B) Detection of β‐Gal expression by IHC in the submandibular glands (A) and sublingual glands (B) of control (Cont) mice and mice treated with metformin (Met) for 8 weeks. Sections were stained with an isotype and anti‐β‐Gal antibody. Representative images of five samples each are shown. The bar graph shows positive areas in sections (*N* = 5) adjusted as described below. (C, D) Detection of ADAM17 expression by IHC in the submandibular glands (C) and sublingual glands (D) of Cont mice and mice treated with Met for 8 weeks. Sections were stained with an isotype and anti‐ADAM17 antibody. Representative images of five samples each are shown. The bar graph shows positive areas in sections (*N* = 5) adjusted as described below. (A, C) Images of submandibular glands were quantified as the positive area (μm^2^) × 10^−2^/field of view (μm^2^). (B, D) Images of sublingual glands were quantified as the positive area (μm^2^)/number of ducts. Bars = 50 μm. Values are presented as means ± SD. **p < 0.01 (the unpaired Student's *t*‐test). (E) Detection of AQP5 and CK19 protein expression in A253 and MSEC. Whole‐cell lysates prepared from these cells were immunoblotted with anti‐AQP5, anti‐CK19, and anti‐GAPDH antibodies. A representative blot is shown. (F) MSEC were treated with the indicated concentrations of Met for 48 h. Whole‐cell lysates prepared from these cells were immunoblotted with anti‐ADAM17 and anti‐GAPDH antibodies. A representative blot is shown. The bar graph shows the integrated signal intensities of the ADAM17/GAPDH ratio. Data represent the mean ± SD of triplicate assays. **p < 0.01 versus untreated cells (0) (Dunnett's multiple comparison test).

Since senescent cells can be visualized by staining with the widely accepted and used marker senescence‐associated beta‐galactosidase (SA‐β‐gal),^20^ we examined the effects of the metformin treatment on cellular senescence in the salivary glands. The metformin treatment significantly decreased SA‐β‐gal expression in serous acinar cells (Figure 3A) and ductal epithelial cells (Figure 3B) of the salivary glands, as demonstrated in other tissues.^29^ ADAM17, also called tumor necrosis factor‐α‐converting enzyme, is expressed in acinar and ductal cells of hSG.^30^ In the present study, its expression was decreased in serous acinar cells (Figure 3C) and ductal epithelial cells (Figure 3D) of the salivary glands of metformin‐treated mice. To confirm the effects of metformin on ADAM17 expression ex vivo, we established MSEC. Although only the AQP5 protein was identified in A253, the AQP5 and CK19 proteins were both detected in MSEC, which are markers of acinar and ductal cells, respectively^31^ (Figure 3E). Moreover, the metformin treatment dose‐dependently decreased ADAM17 expression in MSCE (Figure 3F). These results indicate that the metformin treatment regulated cellular senescence and ADAM17 expression in epithelial cells of the salivary glands.

### 3.4 Effects of the metformin treatment on saliva production and the secretion of sACE2 and sTMPRSS2 in saliva

**FIGURE 4:**
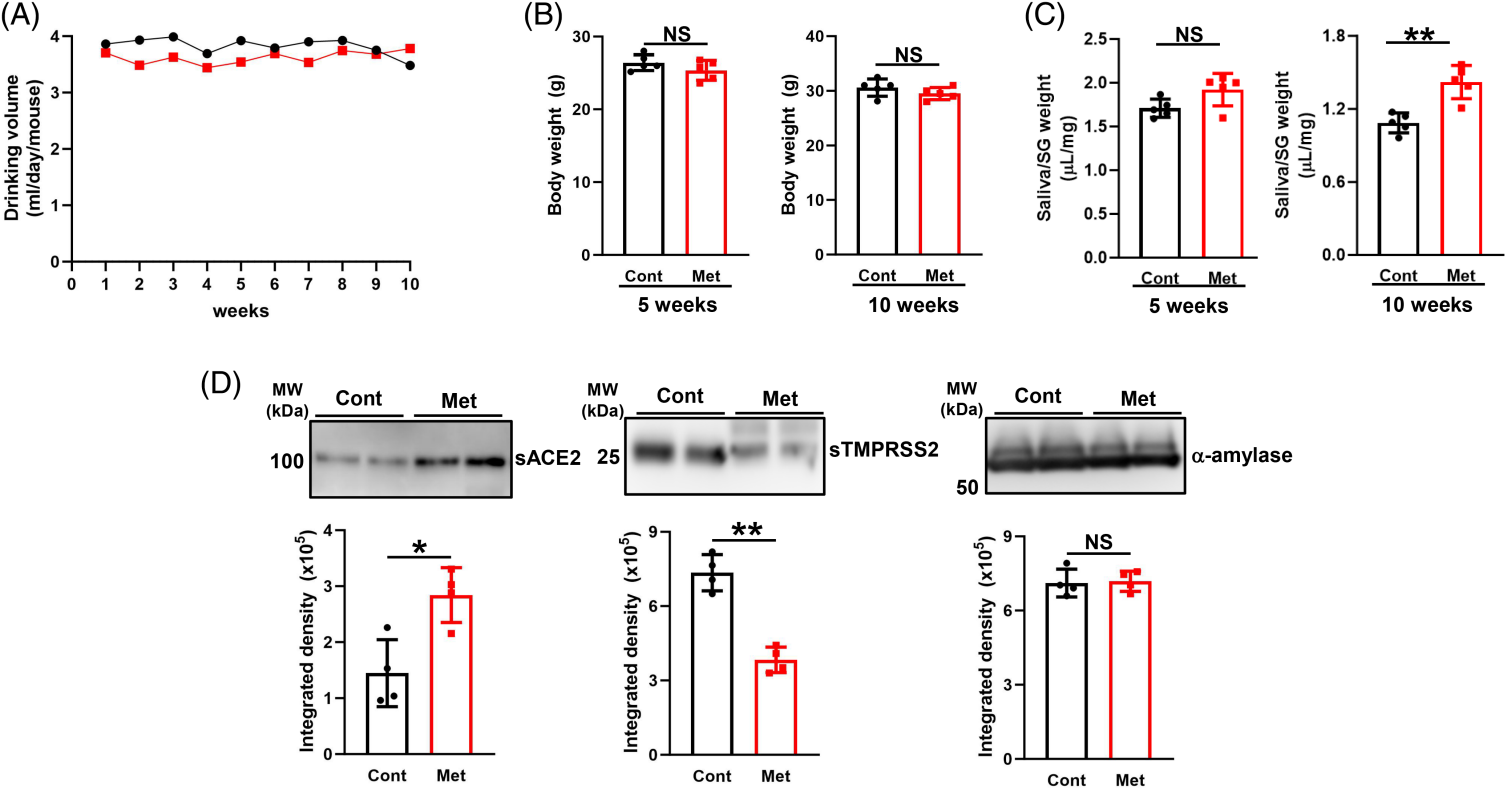
Metformin affects the volume of saliva secreted and sACE2 and sTMPRSS2 levels in saliva. (A) Evaluation of the drinking volume of Cont (water; black line) and Met (metformin, red line) for 10 weeks. Values are presented as means per day per mouse (*N* = 5). (B, C) Body weight (B) and volume of saliva secreted adjusted by the weight of the salivary glands (SG) (C) in control (Cont)‐ and metformin (Met)‐treated mice for the indicated period (*N* = 5 each). (D) Detection of sACE2, sTMPRSS2, and α‐amylase in saliva collected from Cont mice treated and mice treated with Met for 6 weeks by Western blotting and immunoblotted with anti‐ACE2, anti‐TMPRSS2, and α‐amylase antibodies. A representative blot of two independent experiments is shown. The bar graph shows absolute integrated signal intensities (*N* = 4) adjusted to the loading protein amount. (B–D) Values are presented as means ± SD. NS, not significant. **p < 0.01 (the unpaired Student's *t*‐test).

No marked differences were observed in drinking volumes (Figure 4A) or body weights (Figure 4B) between the control and metformin groups over 10 weeks. However, the volume of stimulated saliva was significantly increased in metformin‐treated mice for 10 weeks (Figure 4C). Moreover, although the protein level of α‐amylase, a digestive enzyme of carbohydrates, was not changed, those of sACE2 and sTMPRSS2 were significantly increased and decreased, respectively, in saliva collected from metformin‐treated mice (Figure 4D). Based on the results shown in Figures 1, 2, 3, sACE2 and sTMPRSS2 in saliva appear to be derived from the salivary glands, and metformin may modulate their levels in saliva without affecting their serum levels.

### 3.5 Effects of aging and cellular senescence on the salivary expression of ACE2, TMPRSS2, and ADAM17 in mice

**FIGURE 5:**
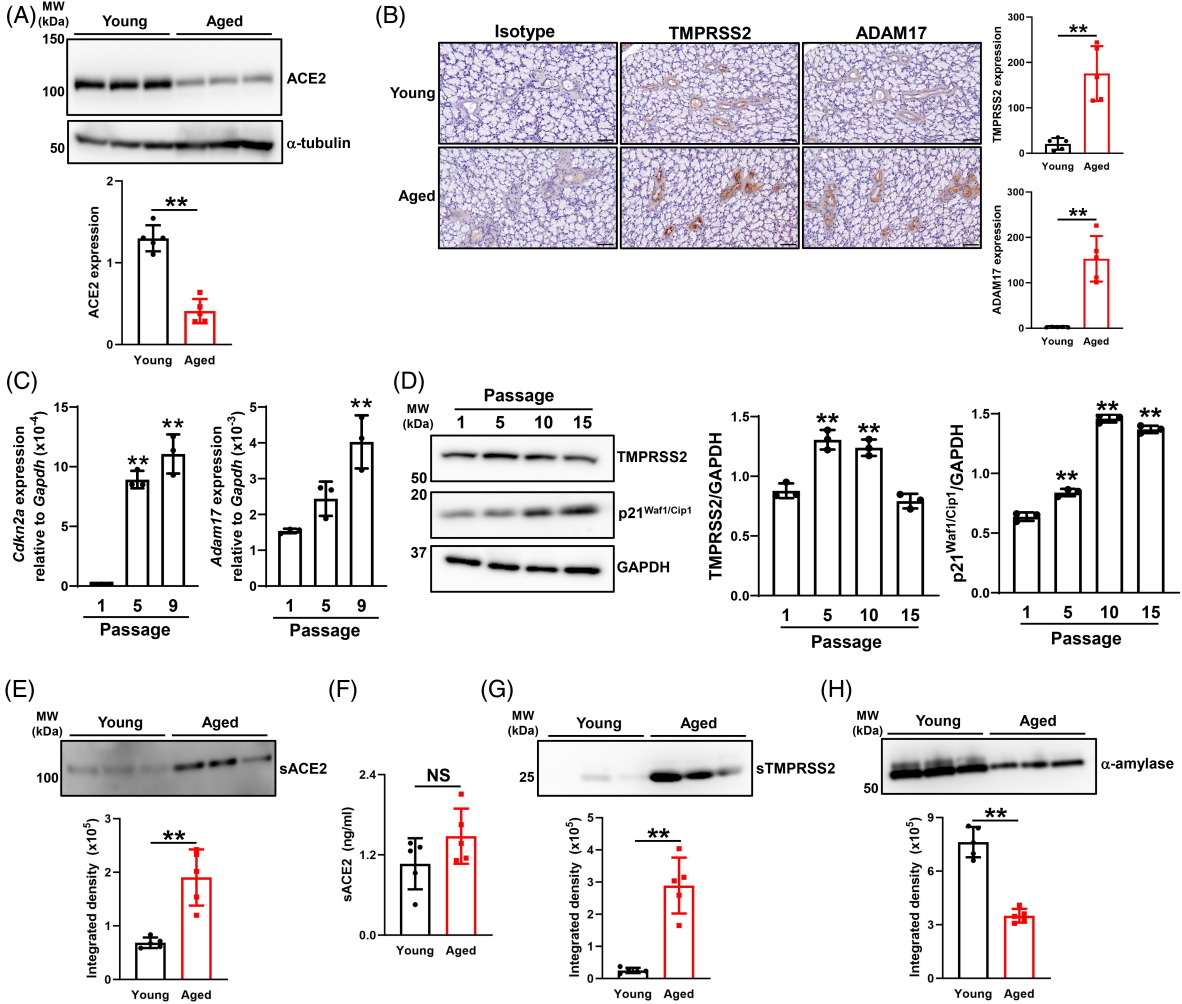
Effects of aging and cellular senescence on the salivary expression of ACE2, TMPRSS2, and ADAM17 in mice. (A) Whole‐cell lysates prepared from the salivary glands (submandibular and sublingual glands) of young and aged mice (*N* = 3 each) were immunoblotted with anti‐ACE2 and anti‐α‐tubulin antibodies. The bar graph shows integrated signal intensities for fACE2 normalized to that of α‐tubulin (*N* = 5). (B) Sublingual gland sections collected from young and aged mice were stained with an isotype, anti‐TMPRSS2, and anti‐ADAM17 antibodies. Representative images of five samples each are shown. The bar graph shows values (*N* = 5) as the positive area (μm^2^)/number of ducts. Bars = 50 μm. (C) MSEC were passaged as indicated (*N* = 3), and *Cdkn2a* and *Adam17* mRNA expression levels were quantified using real‐time PCR. (D) MSEC were passaged as indicated (*N* = 3), and whole‐cell lysates prepared from these cells were immunoblotted with anti‐TMPRSS2, anti‐p21^Waf1/Cip1^, and anti‐GAPDH antibodies. A representative blot is shown. The bar graph shows the integrated signal intensities of the TMPRSS2/GAPDH and p21^Waf1/Cip1^/GAPDH ratio. (E) Detection of sACE2 in saliva collected from young and aged mice (*N* = 3 each) by Western blotting. Immunoblotting with an anti‐ACE2 antibody and a representative blot of two independent experiments is shown. The bar graph shows absolute integrated signal intensities (*N* = 5) adjusted to the loading protein amount. (F) Detection of sACE2 by ELISA in serum collected from young and aged mice (*N* = 5). (G, H) Detection of sTMPRSS2 (G) and α‐amylase (H) in saliva collected from young and aged mice (*N* = 3 each) by Western blotting. Immunoblotting with anti‐TMPRSS2 and α‐amylase antibodies. A representative blot of two independent experiments is shown. The bar graph shows absolute integrated signal intensities (*N* = 5) adjusted to the loading protein amount. Values are presented as means ± SD. NS, not significant. (A, B, E–H) **p < 0.01 (the unpaired Student's *t*‐test). (C, D) **p < 0.01 versus passage 1 (Dunnett's multiple comparison test).

We previously reported the presence of senescent cells in the entire epithelia of the salivary glands of aged mice, particularly ductal cells,^20^ and another study demonstrated that ACE2 positivity in the submandibular glands of humans significantly decreased with increases in age using IHC staining.^32^ Moreover, we confirmed that ACE2 protein levels in the salivary glands were significantly lower in aged mice than in young mice using Western blotting (Figure 5A). On the other hand, TMPRSS2 and ADAM17 expression levels were significantly increased in ductal cells of the salivary glands collected from aged mice (Figure 5B). Moreover, by replicative senescence, the expression of ADAM17 (Figure 5C) and TMPRSS2 (Figure 5D) was significantly increased with the up‐regulation of *Cdkn2a* (*p16* ^*INK4a*^) and p21^Waf1/Cip1^, which are senescence markers,^33^ in MSEC. We^20^ and other researchers^33^ already reported significant decreases in the volume of saliva secreted in aged mice. The present results revealed that sACE2 levels in aged mice were significantly increased in saliva (Figure 5E), but not in serum (Figure 5F), presumably through the up‐regulated expression of ADAM17 in the salivary glands. sTMPRSS2 levels were also significantly increased in the saliva of aged mice (Figure 5G), which was consistent with the increase observed in TMPRSS2 levels in the salivary glands (Figure 5B). However, α‐amylase levels in saliva were significantly decreased in aged mice (Figure 5H). These results indicate that age‐related increases in sACE2 and sTMPRSS2 levels in saliva occurred through the up‐regulated expression of ADAM17 and TMPRSS2, respectively, in the salivary glands.

## 4 DISCUSSION

To the best of our knowledge, this is the first study to show that metformin and aging were involved in the salivary expression of ACE2, TMPRSS2, and ADAM17, which play important roles in the entry of SARS‐CoV‐2 into host cells. Moreover, the metformin treatment significantly increased the volume of saliva secreted without affecting body weight (Figure 4B,C). A previous study reported that metformin improved the accumulation of lymphocytes in the salivary glands, which contributed to the recovery of the salivary flow rate in a mouse model of Sjögren's syndrome (an autoimmune‐related chronic inflammatory disease that typically affects the salivary and lacrimal glands).^34^ However, we herein used wild‐type young mice, in which the accumulation of lymphocytes in the salivary glands was negligible. Since the AMPK activators, AICAR^35^ and adiponectin,^36^ have been shown to increase the volume of saliva secreted by modulating paracellular permeability in the salivary glands of wild‐type rodents, metformin may exert similar effects in acinar cells.

We demonstrated that the metformin treatment significantly increased ACE2 protein expression without affecting its mRNA expression (Figure 1C,E), and salivary ACE2 protein expression in aged mice was significantly decreased (Figure 5A). ACE2 expression was previously reported to be decreased in the lungs of aged rats.^37^ Based on findings showing that the phosphorylation of ACE2 by AMPK enhanced the stability of ACE2,^8^ this stabilizing effect of AMPK may be involved in ACE2 expression in salivary glands. Moreover, since ACE2 has anti‐inflammatory, anti‐fibrosis, and vasodilatory functions^6^ and is a source of sACE2 shed by ADAM17, its stabilizing effect may be beneficial for maintaining homeostasis and preventing SARS‐CoV‐2 infection. On the other hand, Figures 4D and 5E show that sACE2 levels significantly increased in saliva collected from both metformin‐treated mice, though the down‐regulated expression of ADAM17 (Figure 3D,F), and aged mice, presumably via the up‐regulated expression of ADAM17 (Figure 5B,C). Although the pathophysiological roles of sTMPRSS2 in the entry of SARS‐CoV‐2 into host cells currently remain unclear, the present results show significant increases in TMPRSS2 expression in the salivary glands (Figure 5B) and sTMPRSS2 protein levels in saliva (Figure 5G) collected from aged mice indicate the potential of salivary TMPRESS2 as a crucial target for preventing not only SARS‐CoV‐2, but also influenza virus infections, because previous studies demonstrated that TMPRSS2 knock‐out mice were highly tolerant of challenge infections by both SARS‐CoV‐2^38^ and influenza A viruses^39^ in contrast to wild‐type mice.

The cleaved protease domain fragment of TMPRSS2 was detected in lysates collected from the salivary glands (Figure 2B), and its expression was markedly decreased in the salivary glands of metformin‐treated mice (Figure 2C). It was reported that TMPRSS2 was activated via intracellular autocatalysis and this process was blocked in the presence of the hepatocyte growth factor activator inhibitors (HAI)‐1 and HAI‐2,^17^ and HAI‐2 has been shown to alleviate SARS‐CoV‐2 infection.^40^ Although the relationship between metformin and HAI‐2 expression/activity remains unknown, based on the positive effects of metformin on the severity and mortality of COVID‐19,^41^ the inhibition of TMPRSS2 activation cleavage by HAI‐2 may be involved in the effects of metformin.

One limitation of the present study is that we did not provide evidence for the inhibitory effects of sACE2 in saliva on the entry of SARS‐CoV‐2 into host cells. Conventional laboratory strains of mice cannot be efficiently infected with SARS‐CoV‐2 because mouse ACE2 does not support SARS‐CoV‐2 binding.^1^ In future studies, we will examine these inhibitory effects in a mouse‐adapted SARS‐CoV‐2 strain^38^ and human ACE2‐transgenic mice. Since cross‐reactive IgA against the SARS‐CoV‐2S1 subunit^42^ and cationic proteins^43^ in saliva may also exert inhibitory effects, these proteins need to be considered as a valuable strategy to prevent SARS‐CoV‐2 infection and the spread of COVID‐19.

In conclusion, the present results demonstrated that fACE2 and cleaved TMPRSS2 were expressed in the salivary glands, and also that metformin and aging modulated their expression in the salivary glands and sACE2 and sTMPRSS2 levels in saliva. These results provide insights into the mechanisms underlying the effects of metformin and aging on the severity and mortality of COVID‐19, and will encourage further studies on the salivary glands and saliva as potential targets for preventing SARS‐CoV‐2 infection and the spread of COVID‐19.

## AUTHOR CONTRIBUTIONS

Yosuke Shikama, contributed to the conception and design of the study, data acquisition and interpretation, performed all statistical analyses, and drafted and critically revised the manuscript; Kunihiro Otsuka, Masae Furukawa, contributed to data acquisition, drafted and critically revised the manuscript; Yuka Shikama, contributed to data acquisition and analysis, drafted and critically revised the manuscript; Naozumi Ishimaru, Kenji Matsushita, contributed to data interpretation, and drafted and critically revised the manuscript. All authors gave their final approval and agreed to be accountable for all aspects of the work.

## FUNDING INFORMATION

The authors disclosed receipt of the following financial support for the research, authorship, and/or publication of this article: this work was supported by Research Funding for Longevity Sciences from the National Center for Geriatrics and Gerontology (Grant #21‐6 to Kenji Matsushita, #24‐4 to Yosuke Shikama) and JSPS KAKENHI (Grant Number 21H03115 to Yosuke Shikama). The funder had no role in the study design, data collection and analysis, decision to publish, or manuscript preparation.

## CONFLICT OF INTEREST STATEMENT

The authors declare no potential conflicts of interest with respect to the research, authorship, and/or publication of this article.

## Supporting information


**FIGURE S1.** Detection of ACE2 and TMPRSS2 expression by IHC in submandibular glands (SM) and sublingual glands (SL) of young mice. Sections were stained with an isotype and anti‐ACE2 and anti‐TMPRSS2 antibodies. Representative images of five samples each are shown. Bars = 50 μm.

## Data Availability

Raw data were generated at National Center for Geriatrics and Gerontology. Derived data supporting the findings of this study are available from the corresponding author on request.
