# Supplementary material for: Involvement of metformin and aging in salivary expression of ACE2 and TMPRSS2
Source: Biofactors. 2025 Jan 26;51(1):e2154. doi: 10.1002/biof.2154 (PMC11771682; doi:10.1002/biof.2154)
Supplement: Supplementary file 1 — FIGURE S1. Detection of ACE2 and TMPRSS2 expression by IHC in submandibular glands (SM) and sublingual glands (SL) of young mice. Sections were stained with an isotype and anti‐ACE2 and anti‐TMPRSS2 antibodies. Representative images of five samples each are shown. Bars = 50 μm. [file BIOF-51-0-s001.docx]

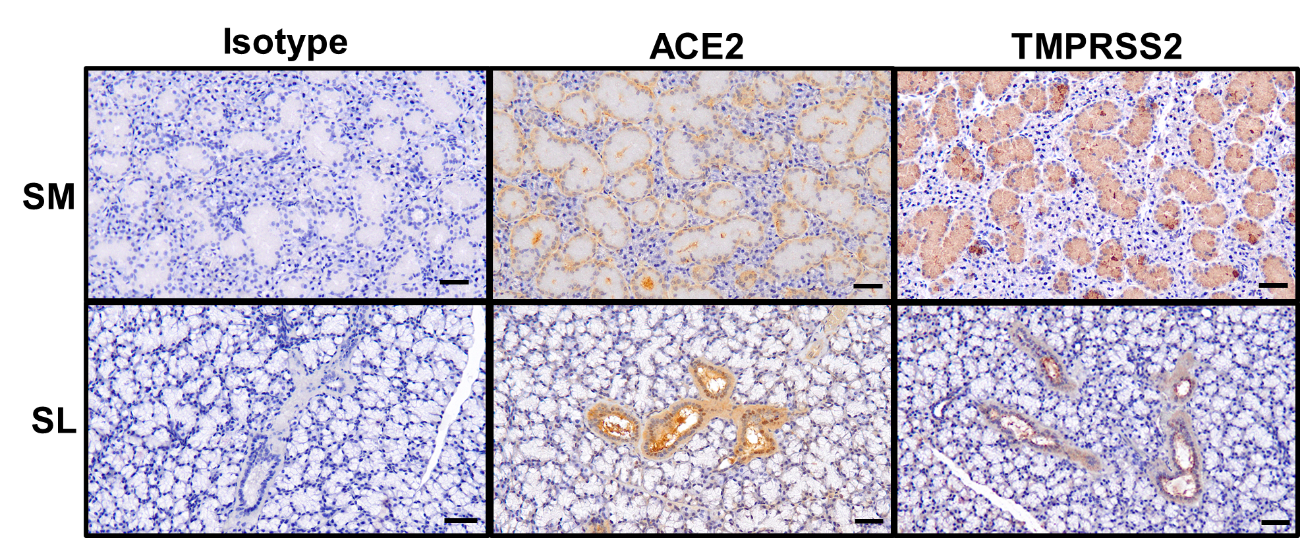


**Supplementary Fig.** Detection of ACE2 and TMPRSS2 expression by IHC in submandibular glands (SM) and sublingual glands (SL) of young mice. Sections were stained with an isotype and anti-ACE2 and anti-TMPRSS17 antibodies. Representative images of 5 samples each are shown. Bars = 50 μm.
